# DREAM, a possible answer to the estrogen paradox of the Women's Health Initiative Trial

**DOI:** 10.1016/j.heliyon.2021.e08666

**Published:** 2021-12-25

**Authors:** Judith C. Hugh, Lacey S.J. Haddon, John Maringa Githaka, Gilbert Bigras, Xiuying Hu, Brittney Madden, John Hanson, Zsolt Gabos, Nadia V. Giannakopoulos, Fleur Huang, Mary M. Hitt, Kirk J. McManus, David Olson, Kelly Dabbs, John R. Mackey

**Affiliations:** a Department of Laboratory Medicine and Pathology, University of Alberta, 116 St & 85 Ave, Edmonton, Alberta T6G 2R3 Canada; b Department of Biochemistry, University of Alberta, 116 St & 85 Ave, Edmonton, Alberta T6G 2R3 Canada; c Department of Psychiatry, University of Alberta, 116 St & 85 Ave, Edmonton, Alberta T6G 2R3 Canada; d Department of Oncology, University of Alberta, 116 St & 85 Ave, Edmonton, Alberta T6G 2R3 Canada; e Department of Oncology, University of Manitoba, 675 McDermot Avenue, Winnipeg, Manitoba R3E 0V9, Canada

**Keywords:** Breast cancer, Estrogen, DREAM, Hormone therapy

## Abstract

Estrogen is thought to cause proliferation of all estrogen receptor positive (ER+) breast cancers. Paradoxically, in the Women's Health Initiative Trial, estrogen-only hormone replacement therapy reduced the incidence and mortality of low grade, ER+, HER2- breast cancer. We gave estradiol to 19 post-menopausal women with newly diagnosed low-grade, ER+, HER2- breast cancer in a prospective window of opportunity clinical trial and examined the changes in proliferation and gene expression before and after estradiol treatment. Ki67 decreased in 13/19 (68%) patients and 8/13 (62%) showed a decrease in Risk of Recurrence Score. We chose three prototypical estrogen responders (greatest decrease in ROR) and non-responders (no/minimal change in ROR) and applied a differential gene expression analysis to develop pre-treatment (PRESTO-30^core^) and post-treatment (PRESTO-45^surg^) gene expression profiles. The PRESTO-30^core^ predicted adjuvant benefit in a published series of tamoxifen, the partial estrogen agonist. Of the 45 genes in the PRESTO-45^surg^, thirty contain the Cell cycle genes Homology Region (CHR) motif that binds the class B multi-vulva complex (MuvB) a member of the DREAM (Dimerization partner, retinoblastoma-like proteins, E2F, MuvB) complex responsible for reversible cell cycle arrest or quiescence. There was also near uniform suppression (89%) of the remaining DREAM genes consistent with estrogen induced activation of the DREAM complex to mediate cell cycle block after a short course of estrogens. To our knowledge, this is the first report to show estrogen modulation of DREAM genes and suggest involvement of DREAM pathway associated quiescence in endocrine responsive post-menopausal ER+ breast cancers.

## Highlights

• WHI trial found that estrogen decreases some ER+ post-menopausal breast cancers.
• Our prospective clinical trial confirmed an anti-proliferative effect of estradiol.
• DREAM pathway genes were downregulated during the anti-proliferative response.
• This suggests that estrogen may activate DREAM quiescence of ER+ breast cancers.

## 1 Introduction

It is generally accepted that estrogen stimulates estrogen receptor (ER) mediated transcription of proliferation genes [1] resulting in the growth of all ER+ breast cancers. Endocrine therapy designed to block the growth effect of estrogen is recommended for all women with ER+ breast cancers and is now the most widely prescribed therapy for patients with cancer [2]. However, the response to estrogen in normal breast cells is not proliferative [3, 4] and the incidence of ER+ breast cancers increase after the menopause when circulating levels of estrogen are lowest [5]. There is also clinical data showing that estrogen produces therapeutic responses in post-menopausal women with breast cancer [6, 7, 8]. More recently, the 20-year follow-up of the Women's Health Initiative (WHI) randomized trial found that women taking estrogen monotherapy as hormone replacement therapy had a decreased incidence and mortality of ER+ breast cancer [9]. This suggests that some post-menopausal ER+ breast cancers respond to estrogen with a paradoxical decrease in proliferation.

The characteristics of the tumours suppressed by estrogen monotherapy in the WHI trial were ER+, HER2-, grade 1 or 2, and node negative [9] consistent with the Luminal A or less aggressive subtype of ER+ breast cancers. Luminal A tumours are also characterized by increased levels of ER, few genetic abnormalities and marked reductions in recurrence with five or more years of adjuvant hormonal therapies [1]. No cell lines exist of the Luminal A subtype which represents 75% of ER+ and 50% of all breast cancers [10] so it is unclear if they proliferate in response to estrogen like the more aggressive Luminal B subtype. We studied tissues from a similar group of post-menopausal women with newly diagnosed ER+ breast cancer in a prospective window of opportunity trial of estrogen therapy. After defining responders who had an estrogen induced decrease in proliferation, we used a bioinformatic approach with differential gene expression profiles to determine a possible mechanism for the estrogen induced anti-proliferative response.

## 2 Materials and methods

**Patients:** All patients were recruited from the Cross Cancer Institute, Edmonton, Alberta Canada. Patients were 55 years of age or older, at least 5 years post menopause without interval exogenous estrogen exposure, low grade, clinically node negative, ER+, HER2 negative, with no contra-indications to estrogen (See Table 1 for clinical characteristics). Estradiol 6mg/day was given for 7–14 days prior to surgery. All patients provided written informed consent. The trial was approved by the Cancer Committee of the Health Research Ethics Board of Alberta (HREBA.CC-14-0169_REN7), received a no-objection letter from Health-Canada and was registered with clinical trials.gov (PRe-operative ESTradiOl Window of Opportunity Study in Post-Menopausal Women with Newly Diagnosed ER Positive Breast Cancer (PRESTO), Trial registration: ClinicalTrials.gov, NCT02238808).

**Table 1:** Clinical Characteristics of the 19 PRESTO patients.

Pt ID	Age	Size	Hist	Gr Bx	Bx-Sx Interval	Base E2	Post E2	Ki67 Bx	Ki67 Sx	ROR Bx	ROR Sx
CCI-002	74.9	1.4	1	2	49	30	994	6.7	6.2	62	52
CCI-003	71.2	1.3	1	2	39	30	1104	2.6	1.7	48	17
CCI-004	69.2	1.2	3	1	37	30	1560	8.9	5.2	ND	32
CCI-005	62.2	1.5	1	1	51	30	575	14.7	10.2	ND	30
CCI-006	60.1	1.1	1	2	42	30	168	4.0	0.6	48	15
CCI-010	67.2	1.4	1	2	35	41	1097	3.0	3.0	39	27
CCI-011	72.4	0.6	2	2	38	76	667	3.3	3.7	ND	33
CCI-012	59.4	2.8	5	1	51	39	470	13.5	5.1	53	53
CCI-013	62.3	0.7	1	1	42	<30	1041	2.4	1.0	63	18
CCI-014	58.3	1.4	1	1	28	<30	284	0.6	1.2	24	24
CCI-015	68.2	1.1	2	2	49	<30	1047	1.7	0.1	ND	37
CCI-016	60.6	0.6	3	1	42	<30	670	4.5	6.9	ND	62
CCI-017	55.4	1.3	5	2	66	<30	839	3.2	1.5	38	31
CCI-018	68.9	0.6	1	1	56	<30	1094	1.3	1.5	17	28
CCI-019	71.0	1.3	1	2	46	<30	817	1.4	0.6	ND	16
CCI-020	65.2	0.9	1	2	35	<30	595	2.1	1.3	48	41
CCI-021	63.0	1.5	1	2	37	46	780	9.6	7.1	50	56
CCI-022	69.7	1.6	2	2	41	57	1184	3.5	3.9	57	60
CCI-023	75.5	1.1	1	2	31	60	1200	4.8	4.5	51	46

**Size**: Tumour size in cm as measured on the surgical specimen. **Hist**: Histologic subtype (surgical): 1 – Invasive Duct NOS, 2 – Invasive Lobular pure, 3 – Mucinous, 5 – Invasive mixed Lobular-Ductal. **Gr Bx**: Overall histologic grade on core biopsy (Scarff-Bloom-Richardson grading system) **Bx-Sx Interval**: the number of days between the core biopsy and surgical procedure. **Base/Post E2:** Pre and Post-treatment serum estrogen (units pg/mL). Detection limit was 30 pg/mL. Any values below this are recorded as <30. **Ki67 Bx/Sx:** Ki67 value by image analysis (Bigras et al. [11]) on core biopsy (Bx) and surgical (Sx) specimen. **ROR Bx**/**Sx**: Risk of Recurrence Score on core biopsy (Bx) and (Sx) surgical specimen. ND – not done on biopsy specimen.

**Outcome:** The study had three goals. The first was to determine if any newly diagnosed ER+ patients had an anti-proliferative response to a short course of estrogens. This outcome was measured by changes in (i) Ki67 by immunohistochemistry using an image analysis protocol [11] and (ii) Risk of Recurrence Score (BC360™, NanoString Technologies Inc., Seattle Washington, USA). Having determined prototypical responders vs. non-responders, we would then look for changes in gene transcription (see RNA-seq below) to develop two Gene Expression Profiles (GEP): a. PRESTO^core^ on the core biopsies which could predict which patients would respond to estrogen and b. PRESTO^surg^ on the surgical (post-treatment) specimens that would highlight differentially altered genes in patients who had experienced an estrogen induced anti-proliferative response. Finally, we would use published Tamoxifen data (a partial estrogen agonist) to test the PRESTO^core^ GEP thereby validating the bioinformatics protocol used to derive the signatures.

**Figure 1:**
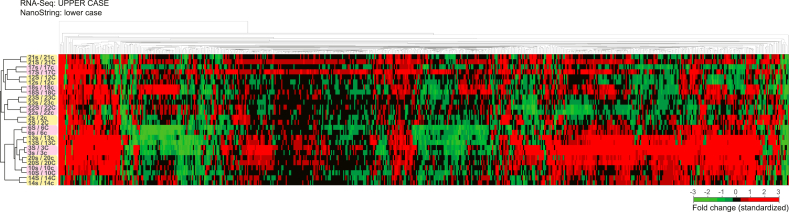
Validation of the RNA-seq data using BC360™. Correlation based unsupervised hierarchical cluster analysis of fold change using BC360™ gene expression data (Nanostring Technologies, Inc. Seattle, Washington, USA) and RNA-seq data. Samples' pairs are highlighted, showing all corresponding patients' consistently cluster together.

**RNA-seq:** Sufficient RNA was extracted from thirteen of 19 core biopsies and all 19 surgical specimens. RNA extracts used for BC360™ analysis were converted to DNA libraries (New England BioLabs, Ontario, CA) and sequenced on the Illumina NextSeq™ 500 system using the NextSeq 500/550 High Output Kit v2.5 (150 Cycles). Paired-end sequencing data were uploaded to the Galaxy web platform and the public server usegalaxy.org [12] was used to align reads to human genome (hg38) using RNA STAR (v2.6.0) [13]. Mapped reads were counted using the featureCounts program (v1.6.3) [14] and differential gene expression analysis was done using the DESeq2 program (v1.18.1) [15]. A heatmap comparing the fold change in RNA-seq to the BC360™ data for the patients' samples (Figure 1) showed an excellent correlation, justifying our use of RNA-seq data in subsequent analysis. All raw sequencing data can be accessed in the NCBI's Gene Expression Omnibus (GEO) database under accession numbers: GSE139688, GSE17705, GSE2990, GSE32222, GSE2034, and GSE20181.

**Identification of Estrogen Response related Profiles:** RNA-seq data from the pre-treatment cores and post-treatment surgical specimens were analyzed separately. We used a random forest approach we recently showed to be more robust than traditional statistical methods in identifying an elusive inflammatory breast cancer signature [16]. Briefly, we filtered for genes with a ≥ 2-fold difference between the responders and non-responders and then classified the two groups in an ensemble of decision trees based bagging, using these genes as predictors. We looped this modeling approach, removing genes with zero or negative predictor importance in each iteration, until all genes left had a positive predictor importance and low out-of-bag error. These were further refined using an iterative process of visual analysis of an unsupervised hierarchical clustering heat map followed by a second random forest analysis filtering.

**Comparison of Gene Signature profiles with published cohorts of ER+ patients:** Original data were downloaded for the validation cohorts from Symmans et al. [17] which included datasets GSE17705 [18] and GSE2990 [19]. All analysis was computed on MATLAB. Datasets were background adjusted and median normalized using the Robust Multi-array Average (RMA) procedure. To determine response scores, PRESTO RNA-seq data (training data) was combined with the validation datasets and quantile normalized to match distribution. Random forest classification was employed where the response score was determined from a sample's score in the trained model. Briefly, a random forest model was trained using the quantile normalized data of the 3 prototypical core responders against the other 10 patients. The resultant model was used to score all the other samples, where response score was fractions of observations of the class per tree leaf, averaged across all trees in the ensemble.

**Identification of transcription factors associated with the PRESTO-45**^**surg**^**profile:** To determine the most common transcription factors associated with the PRESTO-45^surg^ profile, we used the 45 genes as input for transcription factors enrichment analysis, computationally determined by Enrichr [20].

**Identification of FOXM1 binding sites:** ChIP-seq datasets (ENCODE Accession numbers: ENCFF685TME and ENCFF778PWE) obtained through the ENCODE 3 portal [21, 22] on the UCSC genome browser [23] were used to identify regions within 5kb of the transcription start sites (TSS) of the PRESTO-45^surg^ genes where FOXM1 binding has been reported.

**Statistical Analysis:** Absolute Ki67Vv, as well as log transformed Ki67 indices and ROR scores were compared between the core and surgical specimen using a paired *t*-test. A two-tailed p-value was used for significance.

## 3 Results and discussion

**Figure 2:**
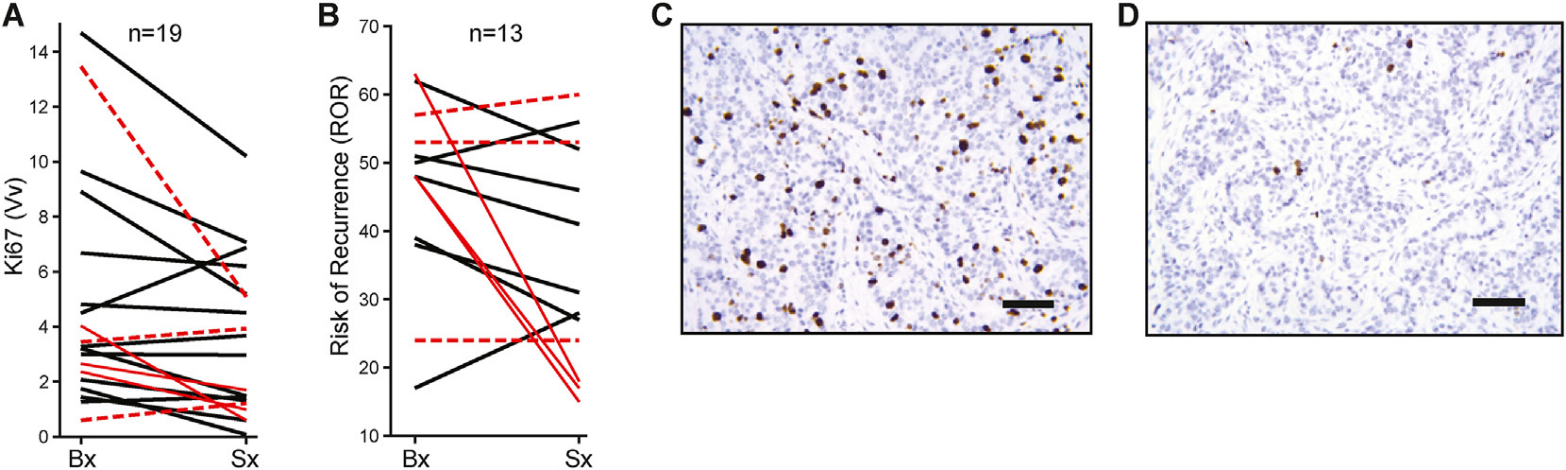
Estrogen decreases Ki67 and Risk of Recurrence (ROR) Score. Changes in Ki67 (A) (*P* = 0.025) and ROR (B) (*P* = 0.07) from the pre-estrogen core biopsy (Bx) to the post-estrogen surgical specimen (Sx) with responders (red solid lines) and non-responders (red dotted lines) shown. Ki67 immunohistochemical (brown) staining for patient CCI-006 before (C) and after (D) estrogen. Scale bar: 100μm.

Nineteen post-menopausal women newly diagnosed with ER+ breast cancer received low dose (6mg/day) estradiol daily for a mean of 12.7 days prior to surgery. Estradiol was well-tolerated with no clinically significant estrogen-related adverse events. Ki67 decreased in 13/19 (68%) patients (Figure 2, A, C, and D) with a percentage change in geometric mean from the pre-treatment core biopsy to the post-treatment surgical specimen of -38.7% (*P =* 0.025). Eight of 13 (62%) patients with sufficient core biopsy material for RNA extraction showed a decrease in ROR (Figure 2B). Although there was a strong correlation between the change in the Ki67 and ROR indices (10/11, kappa = 0.7442), the change in ROR was not significant (*P* = 0.07) due to the smaller number of samples with sufficient core material for RNA-seq.

To define gene expression profiles (GEP) characteristic of an anti-proliferative effect of estrogen, we used a differential expression search strategy to control for the effect of sampling methodology [24] and dichotomized responders vs. non-responders using changes in ROR as the outcome measure because of the known difficulties in Ki67 reproducibility [25]. We defined patients as an estrogen “responder” if they had a reduction in ROR after estrogen treatment that was greater than one standard deviation below the change in ROR score of the total population. This resulted in three patients with the greatest ROR decrease serving as our prototypical estrogen responders (CCI-003, -006, -013, Table 1). The three patients with the least change in ROR between biopsy and surgical were designated as prototypical non-responders (CCI-012,-014, -022).

**Figure 3:**
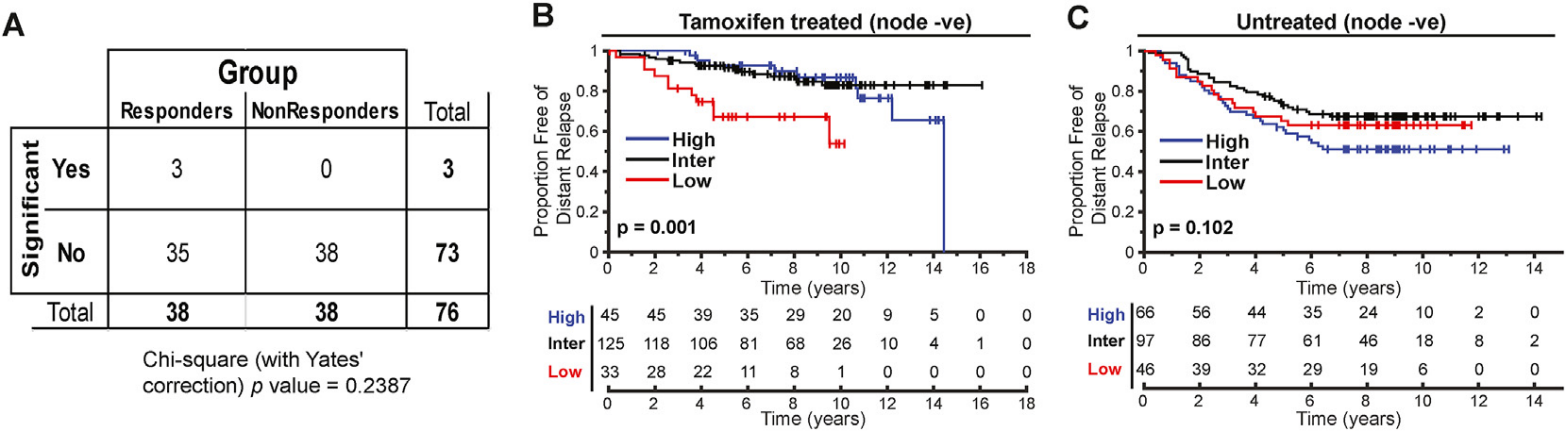
Characteristics of estrogen induced gene transcription changes. (A) Contingency table analysis of the 38 key estrogen induced apoptosis genes (Ariazi et al. [27]) with a log_2_ fold change in responders vs. non-responders (Chi-square test with Yate's correction, P = 0.2383, ns). Kaplan Meier curves for distant relapse free survival for node negative (B) tamoxifen treated (*P* = 0.001) or (C) untreated patients (*P* = ns) scored using PRESTO-30^core^. See text for data sources. Statistical analysis using log-rank. ns – not significant.

We first excluded estrogen induced apoptosis (EIA) due to an endoplasmic reticulum stress response as the mechanism for estrogen's anti-cancer effect [26]. EIA has been observed in tissue cultures exposed to estrogen after prolonged estrogen deprivation. In the current study, no post-estradiol resection specimens showed evidence of apoptotic bodies by light microscopy (data not shown) and comparison of the log_2_ fold change in transcriptomes in responders vs. non-responders before and after estrogen for the 38 genes characteristic of EIA [27] was non-significant (Figure 3A, *P =* 0.24, ns).

We then derived two signatures, a pre-treatment or predictive GEP developed on the core biopsy material and a post-treatment GEP developed from the surgical specimens of differentially expressed genes in the responders compared to the non-responders. This yielded a list of 30 genes in the core biopsies (PRESTO-30^core^, Table 2) and 45 genes in the surgical specimens (PRESTO-45^surg^, Table 3) which accounted for 45% and 73.7% of the variability respectively, in the first principal component of Principal Component Analysis. The PRESTO-30^core^ genes were upregulated in responders and subsequently downregulated after estrogen treatment compared to non-responders. The PRESTO-45^surg^ genes were all downregulated in the responders after estrogen and contained multiple proliferation genes. Each signature could distinguish responders from non-responders only on the sample type (pre vs. post-treatment) from which it was derived. We used the PRESTO-45^surg^ to analyze the post-treatment RNA expression data for the six patients who lacked sufficient core biopsy material for RNA testing and found an additional 3 patients (CCI-004, CCI-015, CCI-019) with gene expression changes consistent with an anti-proliferative response to low dose estrogen for a total of six (32%) responders.

**Table 2:** PRESTO-30^core^ genes.

ADCY10∗	HFE∗	MUC1∗	PPIEL∗	SNORD81
AK5∗	HOXC-AS1	NAALADL2	PRKACB∗	STEAP3-AS1
CATSPER3	ITGB1BP2∗	NAALADL2-AS2	RASD2	SYAP1
CHMP1B2P	KMO∗	NCKAP5	SLC26A4-AS1	TYRP1∗
DNAH11	LOC100289561	NEAT1∗	SLC30A8	ZNF32-AS2
GATA2-AS1	LOC101927708	PKI55∗	SMPDL3A∗	ZNF626

Differentially expressed genes that were upregulated at baseline in the three responders with the greatest estrogen induced decrease in ROR. Asterisks (∗) indicate the 12 genes with data available for comparison with published data (Symmans et al. [17]).

**Table 3:** PRESTO-45^surg^ genes.

**ANLN**	**CENPF**	**ESCO2**	**KIF23**	PRSS8	**TOP2A**
**ASF1B**	**CKAP2L**	**FAM83D**	**KIF2C**	RAB6C	**TPX2**
ASPG	DEGS2	FBXO43	LOC100506474	RAB6C-AS1	**TROAP**
**ASPM**	**DLGAP5**	**HMMR**	LOC728752	RIBC2	**TTK**
**BIRC5**	DSCR9	**IQGAP3**	**MELK**	RRM2	**UBE2C**
**CDC25C**	**DTL**	**KIF14**	**MKI67**	SPATA24	
**CDCA2**	E2F7	**KIF15**	**NCAPG**	**STIL**	
**CENPA**	ERBB3	**KIF18B**	**PBK**	TMEM25	

Differentially expressed genes that were downregulated after estrogen in the three responders with the greatest estrogen induced decrease in ROR. The 30 genes shared between PRESTO-45^surg^ and 268 genes repressed through the p53-LIN37/DREAM pathway (Uxa et al. [32]) are indicated in **BOLD**.

Since tamoxifen has partial estrogen agonist activity, we validated the PRESTO-30^core^ on previously published transcriptome databases of patients treated or untreated with tamoxifen with known clinical follow-up [17]. Only 12 genes of the PRESTO-30^core^ had annotated probe sets in the published database. Despite limiting our search to these 12 genes for which data were available, we found our truncated profile was significantly associated with improved Distant Relapse Free Survival (DRFS) in the tamoxifen treated node negative (Figure 3B*, P* = 0.001) population in the combined MDACC298 (GSE17705) and Sotiriou189 (GSE2990) dataset. There was no association with survival in the untreated cohort (Figure 3C, Wang289 (GSE2034), *n* = 209, *P* = 0.102). This suggests that the PRESTO-30^core^ is a predictive, rather than a prognostic marker, and can identify patients, who will benefit from estrogen or tamoxifen. Importantly, none of the PRESTO-30^core^ genes are proliferation genes included in the meta-PCNA signature which are known to be generically effective at prognostication in ER+ patients [28]. This validates the random forest protocol used to generate the GEP signatures.

We then used the PRESTO-45^surg^ genes as input into the Enrichr program [20] to identify the regulatory elements of the genes that are associated with the sustained estrogen induced decrease in proliferation. The top two transcription factors identified were FOXM1 and E2F4. Further investigation of the PRESTO-45^surg^ genes using published FOXM1 ChIP-seq datasets from the ENCODE 3 portal [21, 22] on the UCSC genome browser [23] showed an enrichment in FOXM1 binding near the TSS for 30 of the 45 genes. The DNA motif (TTTGAA) associated with these peaks was characteristic of the Cell cycle genes Homology Region (CHR) [29].

Both FOXM1 and E2F4 are crucial components of the DREAM-MMB (Dimerization partner, Retinoblastoma-like proteins, E2F4, and MuvB – MYB-MuvB) system of cell cycle control [29]. The system revolves around the ability of the LIN54 member protein of MuvB to stabilize different mutually exclusive complexes on the CHR promoter element of late cell cycle genes. When MuvB is associated with DREAM components, the complex suppresses gene transcription and induces quiescence, a stereotyped form of reversible cell cycle arrest [30]. When MuvB is associated with B-Myb and/or FOXM1 it facilitates activation of the same cell cycle genes (reviewed in [29]). The cell cycle genes regulated by this system include *MKI67* which has a promoter containing two CHR elements [31]. Although the DREAM pathway is characteristically activated after DNA damage through p53 upregulation of p21 transcription [32], hormonal (progesterone) activation of the DREAM pathway was recently shown in an ovarian cancer model [33].

**Figure 4:**
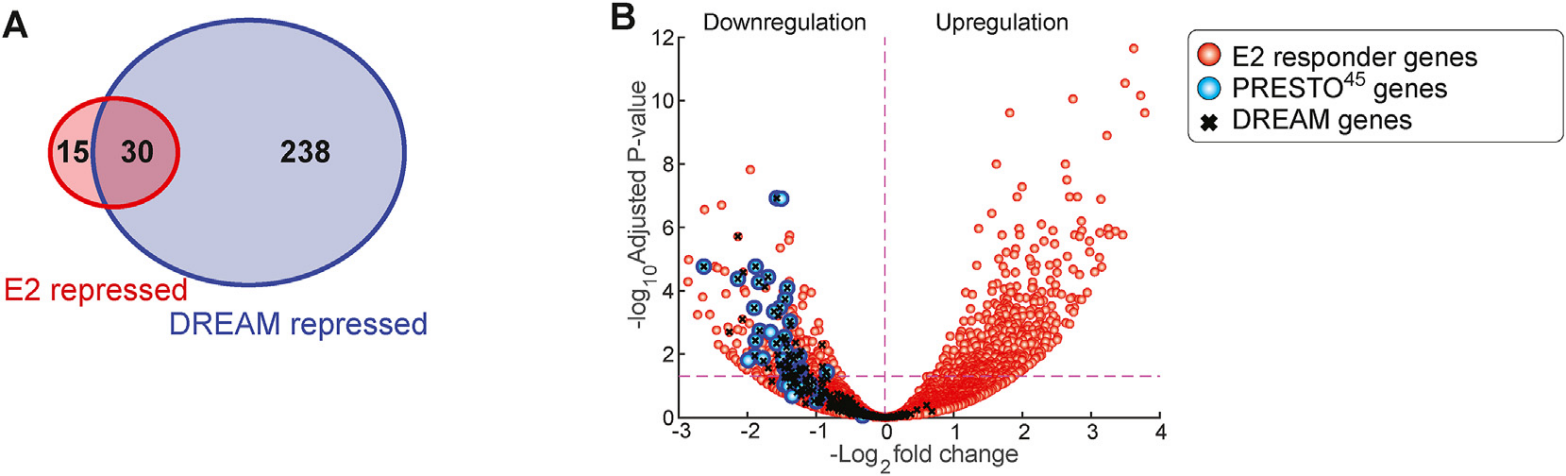
Overlap between PRESTO-45^surg^ and DREAM genes in responders. (A) Venn diagram showing overlap between genes repressed through the p53-LIN37/DREAM pathway (Uxa et al. [32]) and PRESTO-45^surg^. (**B**) Volcano plot of the gene changes in responding patients after estrogen. PRESTO-45^surg^ genes are indicated by blue circles. Genes repressed through the DREAM pathway are marked by an X.

Our findings suggest the possibility that estrogen is also capable of inducing quiescence through DREAM complex binding on the PRESTO-45^surg^ genes, therefore we looked for confirmation that the DREAM pathway had been engaged after estrogen treatment in our prototypical responders. There are 268 genes specifically repressed through the DREAM pathway, identified by having CHR motifs that are activated downstream of p53 and p21 [32]. Thirty of the 45 genes included in the PRESTO-45^surg^ signature are shared with the 268 named genes in the DREAM pathway (Figure 4A) which strongly supports a non-random overlap between the two gene lists (*P* = 8.1199e-50, hypergeometric cumulative distribution function). In addition, two hundred of the 226 (89%) remaining DREAM genes which could be matched in responders were also downregulated (Figure 4B). This near uniform suppression of the DREAM genes is consistent with estrogen induced activation of the DREAM complex to mediate cell cycle block after a short course of estrogens.

Given that short courses of endocrine therapy that cause a decrease in Ki67 predict for long-term response to those agents [34], and Ki67 is a known DREAM regulated gene [31], it is possible that most if not all responses to endocrine therapy in ER+ breast cancers reflect a state of DREAM induced quiescence. This may be a reasonable hypothesis given that endocrine therapy is cytostatic with endocrine responsive tumours having the ability to recur once endocrine treatment is discontinued [35]. In a recently published paper on gene transcription changes after 2 weeks of aromatase inhibitor therapy [36] thirty of the of the first 70 (42.9%) genes significantly regulated by aromatase inhibitors with p < 0.005 are DREAM genes (ref. [36] Supplement Table S11) supporting the hypothesis that the DREAM pathway may be a common therapeutic mechanism in the hormone therapy of ER+ breast cancers. To our knowledge this is the first suggestion that endocrine therapy is inducing cell cycle block mediated through the DREAM pathway in responsive ER+ breast cancers.

**Figure 5:**
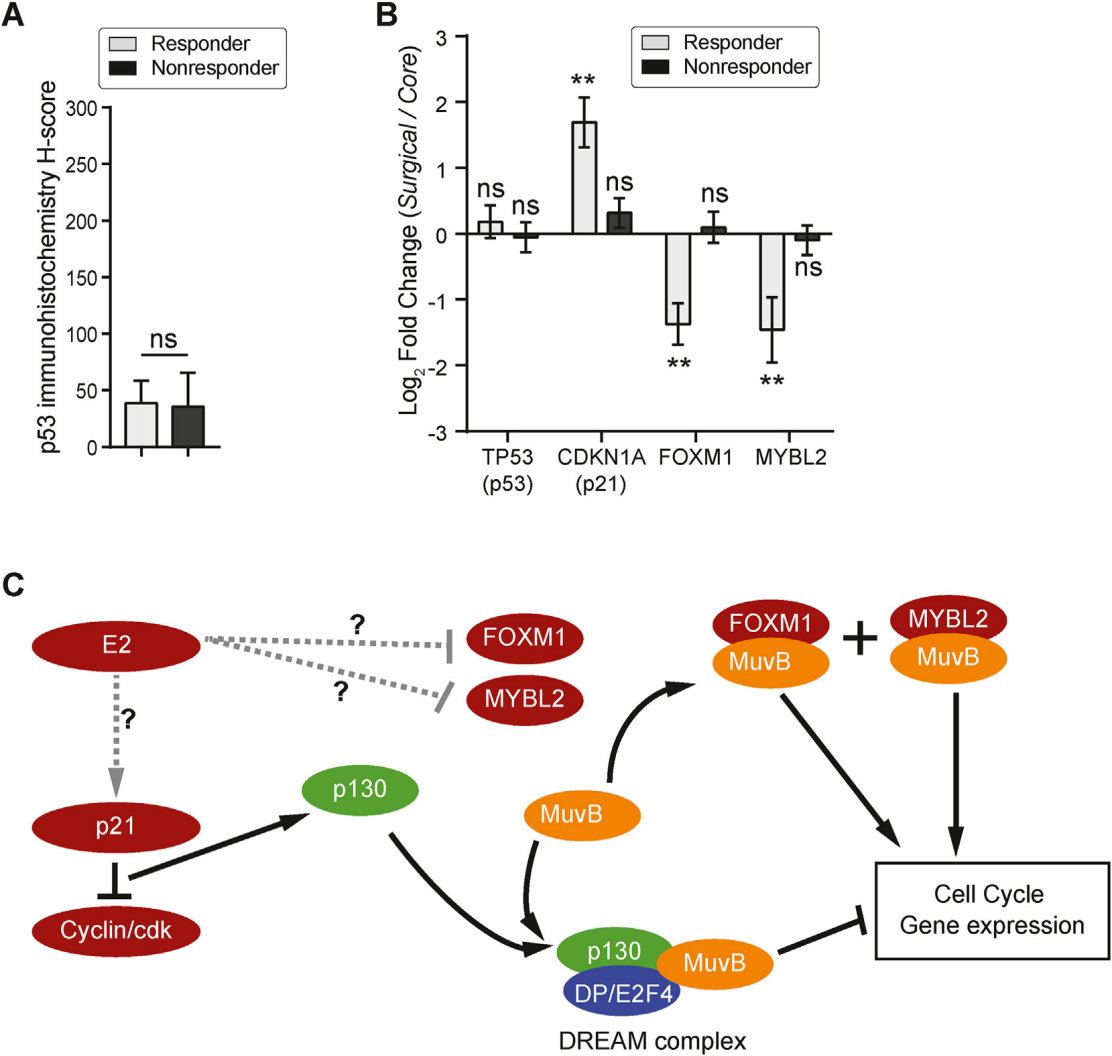
Changes in key DREAM mediators and hypothetical model. (A) p53 protein expression in the surgical specimens of responders and non-responders is similar (p = ns, t-test). p53 protein was detected immunohistochemically using the DO-7 antibody. Expression was calibrated using image analysis and then scored manually using a histoscore calculation (see ref. [42]). (B) Log2 fold change (surgical over core samples) in responders or non-responders for *TP53* (p53), *CDKN1A* (p21), *FOXM1* and *MYBL2,* after estradiol. ∗∗*P* < 0.01, ns – not significant. (C) In this hypothetical model, estrogen therapy is associated with increases in p21 and decreases in FOXM1 and/or MYBL2. The mechanism for these changes in responding patients is currently unknown and this is indicated by dashed lines. Increases in p21 (or cyclin dependent kinase inhibitors, not shown) will lead to hypophosphorylated forms of the retinoblastoma family proteins (RB, p107/RBL1 and p130/RBL2). Hypophosphorylated p130 binds with the LIN52 member of MuvB core proteins resulting in a stable DREAM repressor complex which binds to the CHR element of cell cycle genes through another MuvB core protein (LIN54). The DREAM complex suppresses cell cycle genes causing reversible cell cycle arrest (quiescence). The DREAM complex can be destabilized by increases in pro-proliferative MuvB co-factors, FOXM1 and MYBL2. When MuvB is complexed with these proteins and bound to the CHR motif of the same cell cycle genes, it activates transcription resulting in proliferation.

The DREAM complex is triggered by p21 after p53 activation [32]. We did not find any difference in p53 protein (Figure 5A) or mRNA (Figure 5B) levels between responders vs. non-responders suggesting that if the DREAM pathway has been activated, it is not related to DNA damage or p53. Nevertheless, we found that p21 mRNA (*CDKN1A*) levels were increased by treatment in the 3 prototypical responders (Figure 5B) with no change in the non-responders. The p21 gene (*CDKN1A*) promoter has six Specificity Protein-1 (Sp1) binding sites just upstream to the TATA box. The third Sp1 site has been identified as a binding site for Sp1 bound to the progesterone receptor [37], the androgen receptor [38] and the likely site of an ER-Sp1 complex [39]. Thus, it is possible that estrogen or either of the two other steroid hormones in the absence of estrogen could increase the levels of *CDKN1A* mRNA and the p21 protein. Alternatively, formation of the DREAM complex could be favoured by decreases in the pro-proliferative transcription factor partners for MuvB such as FOXM1 and/or MYBL2 [40]. We compared the estrogen induced transcription changes of the pro-proliferative competitors for the MuvB complex, MYBL2 and FOXM1 in the PRESTO series. Both *FOXM1* and *MYBL2* were significantly decreased by estrogen in responders only (Figure 5B). Although we did not investigate the mechanism(s) whereby estrogen directly or indirectly mediates this decrease, it is possible that estrogen can directly effect the decrease since estrogen receptor response elements (ERE) have been described in the promoter region of FOXM1 [41] and using the ENCODE 3 portal [21, 22], an ERE is present in the vicinity of the progesterone receptor response element responsible for MYBL2 downregulation [33]. Therefore, it is possible that DREAM complex formation can be initiated and/or sustained in responsive patients by hormone induced upregulation of p21 and/or direct or indirect downregulation of the DREAM destabilizing factors FOXM1 or MYBL2. A theoretical schema for estrogen mediated DREAM repression of the cell cycle is presented in Figure 5C. It should be stressed that this paper is hypothesis generating only and considerable work is required to prove DREAM involvement and if confirmed, to uncover the actual mechanism of DREAM regulation in ER+ tumors.

In conclusion, this study confirms that there are post-menopausal ER+ breast tumours that have an anti-proliferative response to a short course of estrogens and suggests that this may be occurring through activation of DREAM associated quiescence. Our findings are consistent with the physiological response of normal breast tissue to estrogen and suggest that low-grade, genetically non-complex ER+ breast cancers share a similar anti-proliferative response to estrogen. This differs from high-grade, genetically complex ER+ breast cancers that have a well-documented and aberrant proliferative response to estrogen.

## Declarations

### Author contribution statement

Judith C. Hugh: Conceived and designed the experiments; Wrote the paper.

Lacey S. J. Haddon: Conceived and designed the experiments; Performed the experiments; Wrote the paper.

John Maringa Githaka: Analyzed and interpreted the data; Wrote the paper.

Gilbert Bigras, John Hanson: Analyzed and interpreted the data.

Xiuying Hu, Brittney Madden, Mary M. Hitt, Kirk J. McManus: Performed the experiments.

Zsolt Gabos, Fleur Huang, David Olson, Kelly Dabbs: Contributed reagents, materials, analysis tools or data.

Nadia V. Giannakopoulos: Conceived and designed the experiments.

John R. Mackey: Conceived and designed the experiments; Contributed reagents, materials, analysis tools or data.

### Funding statement

This work was supported by the Lilian McCullough Chair in Breast Cancer Surgery, J&D Calhoun Memorial Cancer Research Fund, Canadian Breast Cancer Foundation Prairies/NWT (now 10.13039/501100000015Canadian Cancer Society Research Institute, grant # 300278), and the Investigator Initiated Trial Program (10.13039/501100000001Alberta Cancer Foundation, grant #32798).

### Data availability statement

Data associated with this study has been deposited at NCBI Gene Expression Omnibus (GEO) database under the accession number GSE139688, GSE17705, GSE2990, GSE32222, GSE2034, and GSE20181.

### Declaration of interests statement

The authors declare no conflict of interest.

### Additional information

No additional information is available for this paper.
